# Effect of Diet Supplemented With Rapeseed Meal or Hydrolysable Tannins on the Growth, Nutrition, and Intestinal Microbiota in Grass Carp (*Ctenopharyngodon idellus*)

**DOI:** 10.3389/fnut.2019.00154

**Published:** 2019-09-25

**Authors:** Jingting Yao, Peng Chen, Einar Ringø, Gaigai Zhang, Zhongyuan Huang, Xueming Hua

**Affiliations:** ^1^Centre for Research on Environmental Ecology and Fish Nutrition of the Ministry of Agriculture, Shanghai Ocean University, Shanghai, China; ^2^Key Laboratory of Freshwater Aquatic Genetic Resources, Ministry of Agriculture, Shanghai, China; ^3^National Demonstration Center for Experimental Fisheries Science Education, Shanghai Ocean University, Shanghai, China; ^4^Editorial Office of Journal of Shanghai Ocean University, Shanghai, China; ^5^Faculty of Biosciences, Fisheries and Economics, Norwegian College of Fishery Science, UiT The Arctic University of Norway, Tromsø, Norway

**Keywords:** *Ctenopharyngodon idellus*, hydrolysable tannin supplementation, rapeseed meal, metabolism, intestinal microbiota

## Abstract

Grass carp (*Ctenopharyngodon idellus*; *n* = 320) were received four different diets for 56 days. The experimental diets were: fishmeal (FM) containing 10% fishmeal (without rapeseed meal), and rapeseed meal (RM) containing 50% rapeseed meal (without fishmeal), and two semi-purified diets either without (T0) or with 1.25% (T1) supplemental hydrolysable tannin. The approximate content of tannin in the RM diet was 1.31%, which was close to that of T1, while the tannin content of FM was close to that of T0. The weight gain rate of grass carp of the RM group was significantly lower than that of the FM group, while the feeding conversion ratio and the feeding rate were significantly higher in the T1 group than in T0. The muscle lipid content was significantly lower in RM than in FM, while T1 was lower than T0. Intestinal activities of trypsin and α-amylase were significantly higher in T1 and RM groups compared with the other treatments. The hepatic activities of aspartate aminotransferase and alanine aminotransferase were lower in T1 and RM groups compared with the other treatments, while hepatic glycogen, and malonaldehyde were significantly higher in T1 and RM groups. In serum, the total protein and globulin contents were significantly higher in T1 and RM groups, while albumin was significantly lower in the RM group compared to the FM group. High-throughput sequencing showed that Proteobacteria, Firmicutes, and Actinobacteria were the dominant bacterial phyla among groups. The intestinal microbial diversity was higher in T1 and RM. Redundancy analysis showed that tannin, rapeseed meal, and intestinal trypsin activity were positively or negatively correlated with the relative abundance of several different intestinal microbiota at phylum and/or genus levels. The results indicated that 1.25% tannin could not be the main reason for the poor growth of grass carp of the RM group; however, the protein metabolism was disturbed, the absorption of carbohydrate was improved, and the accumulation of lipid had decreased. Furthermore, tannin and rapeseed meal supplementations modulated the intestinal microbiota, and may sequentially regulate the intestinal function by fermenting dietary nutrition, producing digestive enzymes, and modulating probiotics.

## Introduction

High-throughput sequencing technologies enabled enhanced insights into the fish intestinal microbiota, which exert important effects for growth, nutrition, immunity, the maintenance of normal intestinal function, and the resistance against invasive pathogens (1, 2). However, many factors influence the intestinal microbial community, e.g., host, developmental stage, environment, and diet (3–5). Several studies found no dietary effects (6, 7), while others identified clear effects (5, 8, 9). This difference may be due to bacterial classification (autochthonous or allochthonous bacteria), conditions of the intestine (the feeding habits of host, physiological characteristics, and residence time), the utilized molecular methods and the composition of diets (5).

Several studies have identified the relationship between intestinal microbiota and plant protein ingredients, especially for soybean meal (SBM) (10, 11). However, less information is available on the influence of dietary rapeseed meal and rapeseed meal-derived antinutritional factors (ANFs) on intestinal microbiota of aquaculture species (12, 13). A study on the gilthead sea bream (*Sparus aurata*) reported that a replacement of 16% dietary fishmeal with rapeseed meal did not cause a significantly effect on the stomach and intestinal microbiota (14).

Rapeseed meal is an important feed ingredient in aquaculture (15, 16). Due to its high protein content (32–45%, dry matter) and relatively well-balanced amino acid profile, partial replacements of dietary fishmeal with rapeseed meal were investigated in a number of teleost fish species (17, 18). However, negative effects were identified when high levels of rapeseed meal were substituted. Webster et al. (19) reported that 48% dietary rapeseed meal impaired the growth performance of channel catfish (*Ictalurus punctatus*). Bu et al. (20) reported that when 20% fishmeal in the diet of Ussuri catfish (*Pseudobagrus ussuriensis*) was replaced by rapeseed meal, both the antioxidant capacity and non-specific immunity were impaired. Several studies suggested that an unbalanced nutritional profile, high fiber level (132 g/kg dry matter), and ANFs (e.g., glucosinolates, phytic acid, and tannin) would impair nutrition metabolism, negatively affect organ health, and thus decrease growth performance (21–24).

Tannins are important ANFs in rapeseed meal and are secondary compounds with various chemical structures, which can be divided into hydrolysable and condensed tannins (25). *In vivo*, hydrolysable tannins degrade into smaller compounds and injure both liver and kidney (26), which was explored in animals such as ruminants, rodents, and poultry (27, 28). Interestingly, hydrolysable tannins did not induce adverse reactions in pigs, rats, and steers (29, 30). A hydrolysable tannin extracted from chestnut was beneficial for chicken intestinal health by controlling the proliferation of *Clostridium perfringens* at a dietary supplementation level of 1.5–12.0 g/kg (27). A hydrolysable tannin-rich extract reduced intestinal skatole production and optimized the duodenum mucosa morphology of male pigs at a dietary supplementation level of 1–3% (28). Although tannins were accepted to affect digestion by binding proteins or other minerals *in vitro*, the effects of dietary hydrolysable tannins, whatever included in rapeseed meal or supplemented to enhance fish growth performance and health remain unclear, as does its effect on intestinal microbiota.

The grass carp (*Ctenopharyngodon idellus*) is a typical herbivorous fish of central economic importance, and many studies have investigated its growth, immunity, nutrition, and intestinal apoptosis (31–33). Several studies suggested that no more than 35.0% rapeseed meal supplementation in the diet of grass carp would be suitable (34–36). Studies on tannins showed that the Indian major carp could tolerate 2% dietary hydrolysable tannin, while the Nile Tilapia could tolerate 0.5% dietary hydrolysable tannin (37, 38). The present study used the grass carp to characterize intestinal microbiota, growth performance, and several biochemical parameters of the fish diets. Experimental diets contained either 50% rapeseed meal or were supplemented with 1.25% hydrolysable tannin, with the aim to hypothesize the mode of interaction between intestinal microbiota and biological conditions, while providing likely suggestions for the efficient use of rapeseed meal in omnivorous and carnivorous fish diets.

## Materials and Methods

### Experimental Fish and Environmental Conditions

The experiment was conducted at the Shanghai Ocean University coastal aquaculture base located in Binhai, Huinan Town, Pudong New District, Shanghai, China. Grass carp (initial weight: 8.18 ± 0.81 g) were obtained from Huzhou Nanxun Honghao Fisheries. Three hundred and twenty fish were randomly distributed into 16 indoor cages with volumes of 2.5 m^2^ each and 70–80 cm of water depth. Twenty fish were used per cage, and were randomly divided into four groups with four replicates (320 fish total). The fish were fed commercial feed during a 1 week acclimatization period.

Environmental and water quality indicators were monitored daily during the experiment. Cages received filtered pond water and uninterrupted aeration to maintain an appropriate level of dissolved oxygen (>5 mg/L) and ammonium nitrogen (<0.6 mg/L). The water temperature varied between 24 and 32°C.

### Diet and Feeding

The feeding trial lasted for 56 days and all fish were fed to apparent satiation three times a day (7:00 a.m., 12:00 p.m., and 17:00 p.m.). Uneaten diet was collected 30 min after feeding, then dried and weighed for correct the feed intake. During the trial, the feed ration was adjusted when appetite changed. Four isonitrogenous (crude protein 30.6–31.2%) and isoenergetic diets (gross energy 16.8–17.6 MJ/kg) were formulated. Two were practical diets and the further two were semi-purified diets. Practical diets were marked as fishmeal group (FM) and rapeseed meal group (RM), FM with rapeseed meal-free protein sources, containing 10% fish meal, and diet RM containing 50% rapeseed meal without fish meal (Table 1). The semi-purified diets, T0 (0% hydrolysable tannin) and T1 (1.25% hydrolysable tannin), were added casein and gelatin as main protein sources. The approximate percentage content of tannin in the RM diet was 1.31%, which was close to the content in the T1 diet. The approximate content of tannin in the FM diet was 0.16%, close to the content of the T0 diet. All ingredients were grounded into fine powder through a 60-mesh sieve, weighed according to the formula and thoroughly mixed with soybean oil, then water was added and pelleted with an experimental feed mill, dried for about 12 h in a ventilated oven at 40°C. After drying, the diets were broken and sieved into proper pellet size (1.5 mm in diameter), and well stored at a cool dry well-ventilated place.

**Table 1:** Composition and nutrient levels of diets (air dry basis) %.

**Ingredients**	**FM**	**RM**	**T0**	**T1**
Fish meal	10.00	0.00		
Rapeseed meal	0.00	50.00		
Soybean meal	40.00	15.00		
Casein			24.00	24.00
Gelatin			6.00	6.00
Corn starch			17.50	17.50
Rice husk	10.00	10.00		
Wheat middling	26.00	18.00	35.00	35.00
Soybean oil	0.20	0.80	1.40	1.45
Ca(H_2_PO_4_)_2_·H_2_O	2.00	2.00	2.00	2.00
Choline chloride	0.20	0.20	1.00	1.00
Compound vitamins and minerals^a^	1.50	1.50	1.50	1.50
Sodium carboxymethylcellulose	10.10	2.50	11.60	10.30
Hydrolysable tannin^b^			0.00	1.25
**Nutrient levels**				
Crude protein	31.2	30.6	31.0	31.0
Crude fat	3.90	3.80	2.35	2.40
Crude ash	2.48	2.52	4.58	4.40
Moisture	7.83	8.03	8.96	9.42
Cellulose	12.32	9.73	12.20	10.91
Gross energy/(MJ/kg)	17.2	16.8	17.63	17.42
Tannin (%)	0.16	1.31	0.00	1.25

a Compound vitamins and compound minerals were obtained from Hangzhou Haihuang Feed Development Co., Ltd;

b *Hydrolysable tannins was bought from Wuhan Baixing Bio-Technique Co. Ltd, effective substance content is 99%*.

### Sample Collection

At the end of the 56-day feeding period, all fish in each cage were counted and weighed. Seven fish were randomly removed from each cage (28 fish/group), anesthetized with eugenol solution (100 ppm) and killed via sharp blow to the cranium prior to dissection. The exterior of the fish was wiped clean and the abdomen was opened at the ventral mid line. Body weight and intestinal weight were measured. Blood samples were collected from the caudal vein, centrifugation at 4000× g for 10 min at 4°C, removed the serum to centrifuge tubes and stored at −20°C for the detection. Entire intestine, hepatopancreas, and muscle were collected, frozen on ice prior to storage at −20°C. Weight gain (WG), feed conversion ratio (FC ratios), feeding rate (FR), and relative intestine weight were calculated by the following formulae:

WG = (final weight - initial weigh) × 100/(initial weight).
FCR = amount of feed given/bodymass gain.
FR = total feeding × 100/[feeding period × (final weight + initial weight)/2].
Survival rate (%) = final number of fish × 100/initial number of fish.
Relative intestine weight (%) = intestine mass × 100/body mass.

Following the feeding period, three fish were randomly removed from group (collected 5–6 h after the last feeding), anesthetized and killed as described above. The entire intestine tract was removed aseptically and digesta samples (allochthonous bacteria) were collected by carefully squeezing into sterile containers and storage at −80°C prior to DNA extraction.

All procedures were carried out according to national and institutional regulations on the care and use of experimental animals. The experimental handling and treatment of experimental fish were conducted in accordance with the regulations made by the Institutional Animal Care and Use Committee (IACUC), Shanghai Ocean University (SHOU), and this study was approved by the IACUC of SHOU, Shanghai, China.

### Muscle Composition Analysis

Muscle were dried at 70°C for 2 h and then to a constant weight at 105°C to determine the moisture content; protein was determined by measuring nitrogen (N × 6.25) using the Kjeldahl method; lipid by ether extraction using chloroform-methanol extraction, and dry matter by combustion at 550°C (39).

### Parameters and Digestive Enzymes Activities

Hepatopancreas and intestinal samples were homogenized by adding sterile 0.85% saline solution to prepare 10% (W:V) homogenates, and then centrifuged at 4000× g for 10 min at 4°C. Supernatants were used for activities analyze of digestive and immune enzyme in 12 h.

In hepatopancreas, the activities of alanine aminotransferase (ALT) and aspartate aminotransferase (AST), content of malonaldehyde (MDA), and hepatic glycogen were measured using specific analytical procedures and commercially available kits (Jiancheng Bioengineering Institute, Nanjing, China). Serum parameters (total protein, albumin, and urea nitrogen) were measured using specific analytical procedures and commercially available kits (Jiancheng Bioengineering Institute, Nanjing, China).

The intestinal activity of trypsin was analyzed following the method of Natalia et al. (40). The activity of α-amylase was measured according to Worthington (41). The activity of lipase was assayed based on measurement of fatty acids released due to enzymatic hydrolysis of triglycerides in a stabilized emulsion of olive oil (42) using specific analytical procedures and commercially available kits (Jiancheng Bioengineering Institute, Nanjing, China). All enzyme assays were performed in quadruplicate.

### Analyze of Intestinal Microbiota

#### DNA Extraction

DNA was extracted from 200 mg of each digesta sample. DNA extraction was performed using the FastDNA Spin Kit for Soil (MP Biologicals, Solon, OH, USA) according to the manufacturer's specification. DNA concentrations were determined using NanoDrop^TM^ 1,000 spectrophotometer (Thermo Scientific, DE, USA).

#### PCR Amplification

To analyze the bacterial community, amplification of the variable region V3-V4 of the 16S rRNA gene was performed. The PCR was conducted using the bacterial universal primers 338F (5′ ACT CCT ACG GGA GGC AGC AG 3′) and 806R (5′ GGA CTA CHV GGG TWT CTA AT 3′). The PCR reaction was based on Xiong et al. (43). The resulted PCR products were extracted from a 2% agarose gel and further purified using the AxyPrep DNA Gel Extraction Kit (Axygen Biosciences, Union City, CA, USA) and quantified using QuantiFluor™ -ST (Promega, USA) according to the manufacturer's protocol.

#### High-Throughput Sequencing

Purified amplicons were pooled in equimolar and paired-end sequenced (2 × 300) on an Illumina MiSeq platform (Illumina, San Diego,USA) according to the standard protocols by Majorbio Bio-Pharm Technology Co. Ltd. (Shanghai, China). Raw fastq files were quality-filtered by Trimmomatic and merged by FLASH with the following criteria: (i) The reads were truncated at any site receiving an average quality score <20 over a 50 bp sliding window. (ii) Sequences whose overlap being longer than 10 bp were merged according to their overlap with mismatch no more than 2 bp. (iii)Sequences of each sample were separated according to barcodes (exactly matching) and Primers (allowing 2 nucleotide mismatching), and reads containing ambiguous bases were removed.

The library construction and sequencing of bacterial 16S rRNA V3-V4 region were performed by Majorbio Bio-Pharm Technology Co. Ltd. (Shanghai, China).

#### Data Analysis

Data, other than intestinal microbiota, were presented as means ± SD and analyzed via one-way analysis of variance (ANOVA) in SPSS version 17.0. Differences in mean values were analyzed via Duncan's multiple range test when ANOVA identified differences among groups. The level of significance was set to *P* < 0.05.

The data of intestinal microbiota were analyzed on the free online platform of Majorbio I-Sanger Cloud Platform (https://www.i-sanger.com/). Operational taxonomic units (OTUs) were clustered with 97% similarity cutoff using UPARSE(version 7.1) with a novel “greedy” algorithm that performs chimera filtering and OTU clustering simultaneously. The taxonomy of each 16S rRNA gene sequence was analyzed by RDP Classifier algorithm (http://rdp.cme.msu.edu/) against the Silva (SSU123) 16S rRNA database using confidence threshold of 70%. Chimeras and tags with ambiguous bases (N) were excluded for the OTU picking. The corresponding rarefaction curve and heatmap figures were produced with software R project (v3.0.3). Alpha diversity was applied for analyzing the complexity of species diversity among samples using OTUs, Chao, Ace (abundance-based coverage estimator), Shannon, and Simpson indices. The principal component analysis (PCA) of the fast UniFrac metric matrix was used to compare the microflora according to phylogenetic information (44). Kruskal–Wallis rank sum test was used for comparison between FM and RM groups, and T0 and T1 groups, respectively. Differences were regarded as significant when *P* < 0.05.

## Results

### Survival and Growth Performance

The survival rate of FM and T1 were higher than the other treatments (*P* = 0.494), while the relative intestine weight showed opposite trend (*P* = 0.682); however, the weight gain rate of the FM group was significantly (*P* = 0.000) higher than that of the RM group, and was lowest in T0 and T1 groups (Table 2). FC ratios and feeding rates were significantly (*P* = 0.000) higher in the T1 group compared to the T0 group. Weight gain rate, FC ratios, and feeding rate changed with tannin content and followed the same trend between practical feed and semi-purified feed.

**Table 2:** Survival and growth performance of grass carp (means ± SD, *n* = 4).

	**FM**	**RM**	**T0**	**T1**	***p*-value**
Growth					
Survival rate%	95.00 ± 5.78	93.75 ± 4.79	96.25 ± 4.79	98.75 ± 2.5	0.494
Weight gain rate%	549.16 ± 18.58^a^	496.97 ± 18.58^b^	230.96 ± 14.90^c^	228.01 ± 20.22^c^	0.000
Feed conversion ratio	1.21 ± 0.04^c^	1.26 ± 0.03^c^	1.87 ± 0.11^b^	2.04 ± 0.07^a^	0.000
Feeding rate%	3.17 ± 0.11^c^	3.22 ± 0.09^c^	3.57 ± 0.14^b^	3.84 ± 0.15^a^	0.000
Relative intestine weight%	3.47 ± 0.41	3.52 ± 0.51	3.62 ± 0.50	3.46 ± 0.38	0.682
Muscle					
Moisture%	78.16 ± 0.10	78.49 ± 0.12	78.98 ± 0.71	79.29 ± 0.13	0.430
Ash %	1.06 ± 0.04^b^	1.26 ± 0.04^a^	1.26 ± 0.03^a^	1.25 ± 0.04^a^	0.002
Protein%	18.80 ± 0.03^a^	18.42 ± 0.02^a^	17.75 ± 0.03^b^	17.39 ± 0.11^b^	0.000
Lipid%	2.42 ± 0.16^a^	1.85 ± 0.11^c^	2.12 ± 0.13^b^	1.62 ± 0.05^c^	0.000
Intestine					
Trypsin (Umg^−1^ protein)	4597.93 ± 117.75^b^	6942.87 ± 373.27^a^	2503.95 ± 49.82^d^	3078.11 ± 154.12^c^	0.000
α-Amylase (Umg^−1^ protein)	17.95 ± 0.07^c^	20.11 ± 0.06^a^	17.10 ± 0.12^d^	18.99 ± 0.15^b^	0.000
Lipase (Umg^−1^ protein)	24.18 ± 3.04^a^	13.67 ± 0.76^b^	4.71 ± 1.05^c^	2.10 ± 0.10^c^	0.000
Hepatopancreas					
ALT (U/mg)	82.30 ± 3.69^c^	56.51 ± 3.81^d^	122.66 ± 1.78^a^	93.92 ± 2.34^b^	0.011
AST (U/mg)	25.97 ± 1.56^b^	22.90 ± 2.08^b^	30.57 ± 1.99^a^	24.12 ± 1.84^b^	0.005
Hepatic glycogen (mg/g)	2.56 ± 0.14^d^	3.07 ± 0.09^c^	5.08 ± 0.07^b^	7.82 ± 0.18^a^	0.000
MDA (nmol/mg prot)	16.46 ± 1.03^c^	32.87 ± 4.98^b^	19.06 ± 2.14^c^	75.69 ± 1.78^a^	0.000
Serum					
Total protein (g/L)	43.00 ± 3.11^b^	54.56 ± 2.25^a^	44.38 ± 0.65^b^	53.01 ± 1.55^a^	0.000
Globulin (g/L)	19.15 ± 3.03^b^	34.80 ± 2.24^a^	26.85 ± 0.49^b^	36.32 ± 2.64^a^	0.000
Albumin (g/L)	23.85 ± 1.28^a^	19.76 ± 0.16^b^	17.53 ± 0.16^c^	16.69 ± 1.13^c^	0.000
Urea nitrogen (mmol/L)	3.77 ± 0.02^b^	2.97 ± 0.06^d^	6.18 ± 0.15^a^	3.40 ± 0.10^c^	0.000

*In the same line, different letters indicate significant differences among four groups (P < 0.05), and the letter in lower order indicated low value; values with no letter mean no significant difference (P > 0.05)*.

### Muscle Composition

The muscle moisture of RM and T1 were higher than the other treatments (*P* = 0.430, Table 2). The muscle ash of the FM group was significantly (*P* = 0.002) lower than that of other groups. The protein contents of FM and RM groups were significantly (*P* = 0.000) higher than those of T0 and T1 groups. FM and T0 groups were higher than RM and T1 groups. The lipid contents of FM and T0 groups were significantly (*P* = 0.000) higher than those of other treatments.

### Digestive Enzyme Activity

The activities of trypsin and α-amylase were significantly (*P* = 0.000) lower in FM and T0 groups compared to the other treatment groups (Table 2). Lipase activity was significantly (*P* = 0.000) higher in the FM group compare to the RM group and was lowest in T0 and T1 groups.

### Hepatopancreas and Serum Biochemical Parameters

In the hepatopancreas, the content of MDA and glycogen were significantly (*P* = 0.000) lower in T0 and FM groups compared to the other treatments, in contrast to the activity of ALT (*P* = 0.011). The activity of AST was significantly (*P* = 0.005) higher in T0 compared to other groups (Table 2). Serum parameters showed that total protein (*P* = 0.000) and globulin (*P* = 0.000) were significantly higher in T1 and RM groups compared to the other treatments, while urea nitrogen was lower in T1 and RM groups. Albumin was significantly (*P* = 0.000) higher in the FM group than in the RM group.

### Characteristics of the High-Throughput Sequence Data and Alpha Diversity Results

The total number of operational taxonomic units (OTUs) assigned from the 12 samples of the studied compartments were 2,067, showing 97% sequence identity. Rarefaction curves (Figure S1) showed that samples reached the saturation phase, indicating adequate sequencing depth.

**Figure 1:**
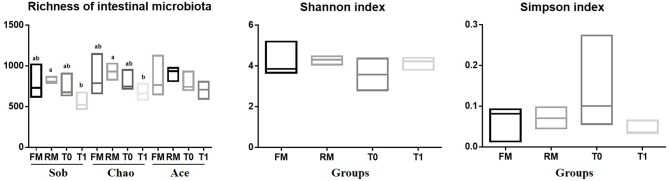
Alpha diversity results of intestinal digesta microbiota of grass carp. The richness and diversity of the microbial community in a single sample were revealed by α-diversity analysis, different letters indicate significant differences among four groups (*P* < 0.05); values with no letter mean no significant difference (*P* > 0.05).

The α-diversity of the allochthonous intestinal microbiota of the grass carp are listed in Figure 1. The richness estimates were supported by sobs, ace, and chao indexes, which refer to the number of bacterial species within a community without considering the abundance of each species. The diversity indexes were supported by the Shannon index and the Simpson index, and were influenced by species richness and species evenness in the sample. The Sob in RM was higher than T1 (*P* = 0.046). The Chao in RM was higher than T1 (*P* = 0.048). The Ace in RM was higher than FM (*P* = 0.454). The Shannon index was higher in the RM and T1 groups compared to the other treatments (*P* = 0.490), and Simpson index was opposite (*P* = 0.311). Some indices indicated no significant difference, which might be attributed to limited samples, intra individual-, and intra-group variations.

### Composition, Abundance, and Difference of Intestinal Microbiota

**Figure 2:**
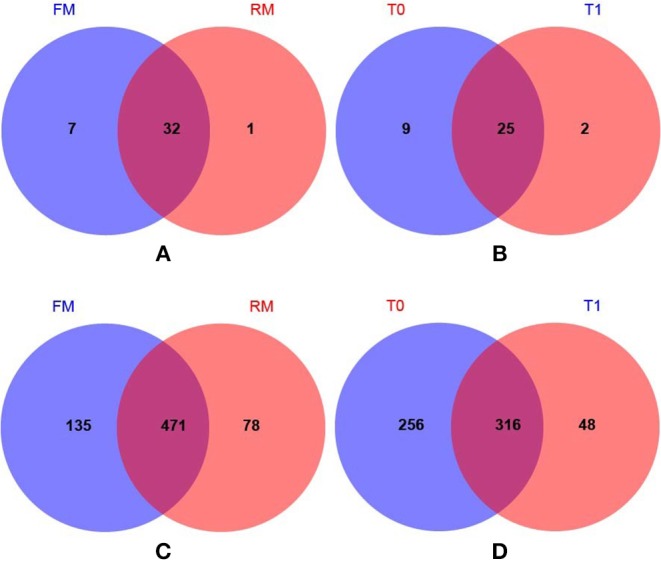
Venn diagrams showed compartmental core microbiota OTU distributions. **(A)** Practical-diet groups: 32 OTUs were identified as core microbiota for the FM and RM group at phylum level. **(B)** Semi-purified-diet groups: 25 OTUs were identified as core microbiota (80% of samples in each compartment) for the T0 and T1 group at phylum level. **(C)** Practical-diet groups: 471 OTUs were identified as core microbiota (80% of samples in each compartment) for the FM and RM group at genus level. **(D)** Semi-purified-diet groups: 316 OTUs were identified as core microbiota (80% of samples in each compartment) for the T0 and T1 group at genus level.

The number of common/unique OTUs were visually displayed using Venn diagrams; the average phylum numbers of FM, RM, T0, and T1 groups were 39, 33, 34, and 27, respectively (Figures 2A,B). The average genus numbers of FM, RM, T0, and T1 groups were 606, 549, 572, and 364, respectively (Figures 2C,D).

**Figure 3:**
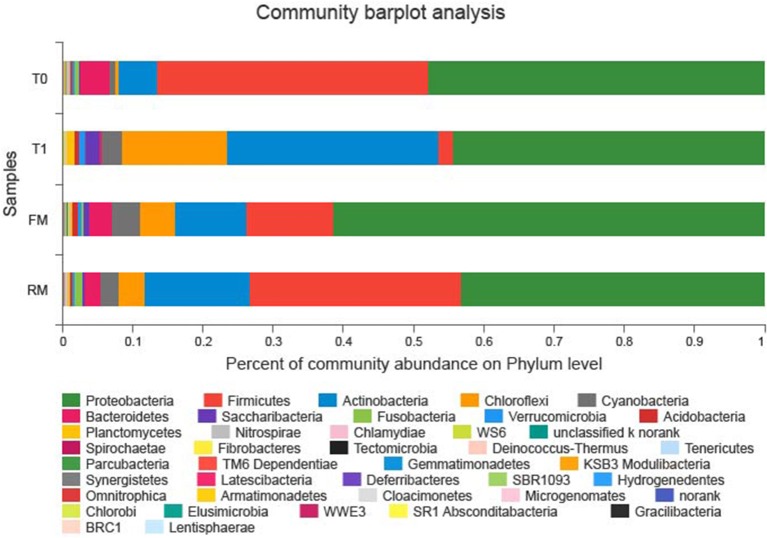
Intestinal bacterial composition (relative OTU composition) at phylum level.

At the phylum level, phyla with the highest relative abundance in each group were ranked and their structures were analyzed and are presented in Figure 3. These included Proteobacteria (FM, 61.73%; RM, 43.4%; T0, 47.31%; T1, 43.99%), Firmicutes (FM, 12.22%; RM, 29.93%; T0, 39.21%; T1, 1.93%), Actinobacteria (FM, 10.13%; RM, 14.95%; T0, 5.55%; T1, 30.68%), Bacteroidetes (FM, 3.18%; RM, 2.15%; T0, 4.19%; T1, 0.24%), Chloroflexi (FM, 4.93%; RM, 3.70%; T0, 0.42%; T1, 14.88%), Cyanobacteria (FM, 4.06%; RM, 2.60%; T0, 0.74%; T1, 3.03%), Saccharibacteria (FM, 0.75%; RM, 0.34%; T0, 0.1%; T1, 2.11%), Fusobacteria (FM, 0.36%; RM, 1.11%; T0, 0.67%; T1, 0.06%). These results indicated that the dominant phyla in the allochthonous intestinal microbiota of the grass carp were Proteobacteria, Firmicutes, and Actinobacteria.

**Figure 4:**
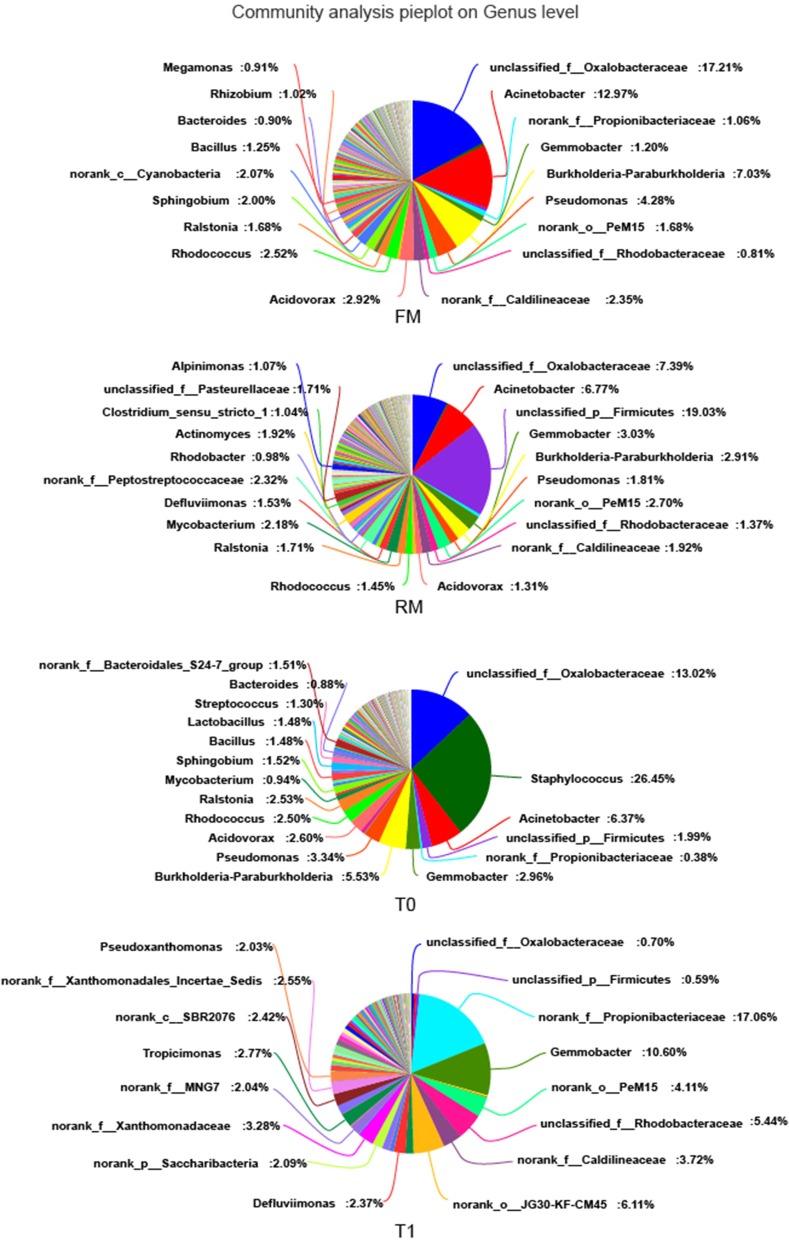
Intestinal bacterial composition (relative OTU composition) at genera level.

The main allochthonous genera are displayed in Figure 4. The genera *Oxalobacteraceae* (17.21%), *Acinetobacter* (12.97%), *Paraburkholderia* (7.03%), and *Pseudomonas* (4.28%) showed the highest abundance in the digesta of the FM group. The genera unclassified_p_Firmicutes (19.03%), *Oxalobacteraceae* (7.39%), *Acinetobacter* (6.77%), and *Gemmobacter* (3.03%) showed the highest abundance in the RM group. In the digesta of the T0 group, genera *Staphylococcus* (26.45%), *Oxalobacteraceae* (13.02%), *Acinetobacter* (6.37%), and *Paraburkholderia* (5.53%) showed the highest abundance while in T1, the genera *Propionibacteriaceae* (17.06%), *Gemmobacter* (10.60%), JG30-KF-CM45 (6.11%), and *Rhodobacteraceae* (5.44%) showed the highest abundance.

**Figure 5:**
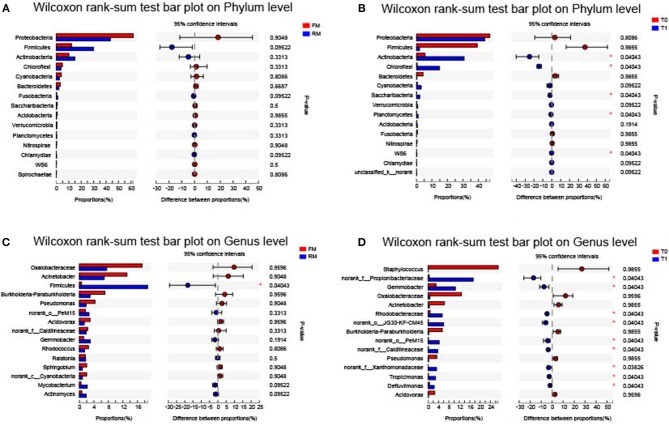
Intestinal bacterial composition differences at phylum and genus level. Data of different groups were showed as relative abundance (%) of phylum **(A,B)** and genus **(C,D)**. **(A)** The bacterial composition differences at phylum in practical-diet groups. **(B)** The bacterial composition differences at phylum in purified-diet groups. **(C)** The bacterial composition differences at genus in practical-diet groups. **(D)** The bacterial composition differences at genus in purified-diet groups. Statistical analysis was performed by the wilcoxon rank-sum test, the “*” indicated significant difference (*P* < 0.05).

The relative levels of allochthonous microbiota between different groups with regard to phylum and genus levels showed the following results: At the phylum level, no significant difference was obtained between FM and RM (Figure 5A), while the relative abundance of Actinobacteria, Chloroflexi, Saccharibacteria, Planctomycetes, and WS6 were significantly (*P* < 0.05) higher in the T1 group than in the T0 group (Figure 5B). At the genus level, the relative abundances of *Propionibacteriaceae, Gemmobacter, Rhodobacteraceae*, JG30-KF-CM45, PeM15, *Caldilineaceae, Xanthomonadaceae, Tropicimonas*, and *Defluviimonas* were significantly (*P* < 0.05) higher in group T1 than in group T0 (Figure 5D). In the RM group, unclassified_p_Firmicutes was significantly (*P* < 0.05) higher than in the FM group (Figure 5C).

**Figure 6:**
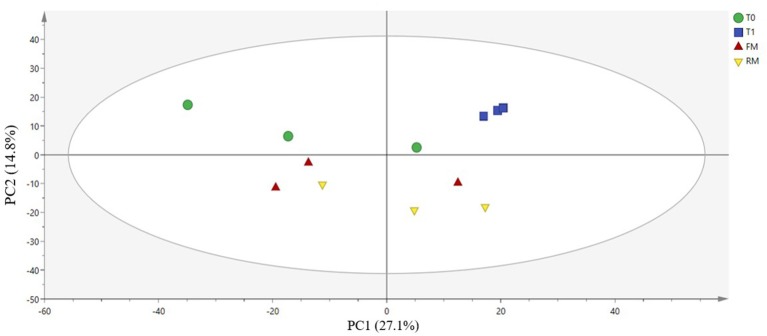
Principal coordinates analysis (PCA) on genus level: scatter plot of PCA indicating variance of fingerprints derived from different microflora. The 1st principal component and the 2nd principal component (PC1, X-axis and PC2, Y-axis) were 27.1 and 14.8%, Q^2^ = 0.0934.

Principal component analysis (PCA) is a method to analyze and simplify data sets. A closer distance between different samples on the PCA map indicates a more similar microbiota composition of the samples. The contribution rates of the first and the second principal component were 27.1 and 14.8%, respectively (Figure 6, *p* = 0.181). All of the samples in the score plots were within the 95% Hotelling's T-squared ellipse, which indicated that no outlier was present among the analyzed samples. Most of the T0 group were centered in the first and second quadrants, while T1 centered in the first quadrant. The samples of the FM group partial overlapped with the RM group, which were distributed throughout the third and fourth quadrants. As indicated, the difference between the T0 and T1 groups was large, while the difference between the FM and RM group was small.

**Figure 7:**
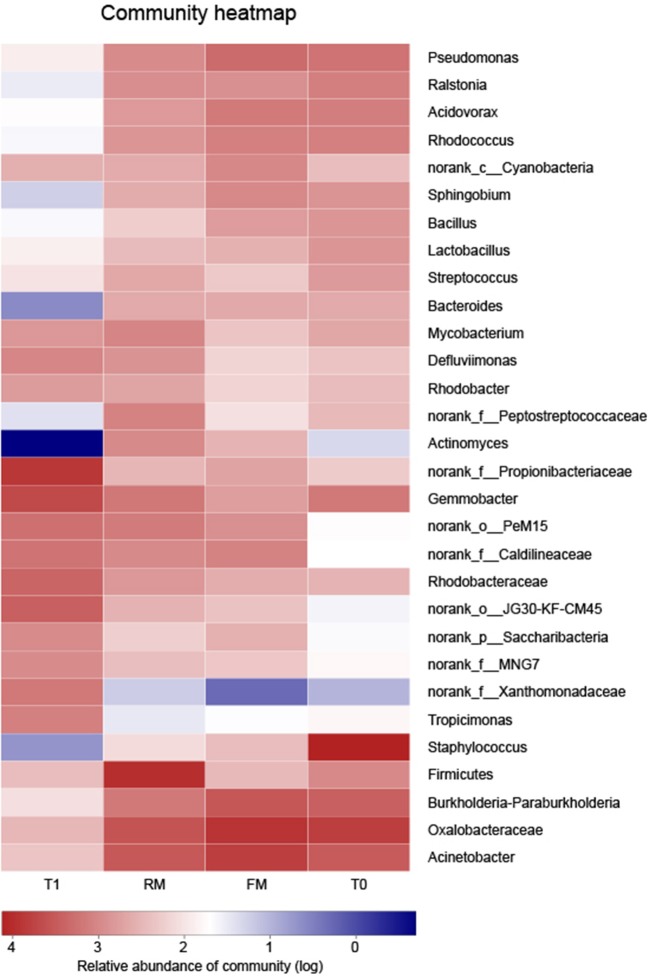
Bacterial distribution of genus level among 4 groups. Rows depict the 30 main bacteria at genus level, columns depict 4 groups. At the low right corner of picture, the color intensity with the legend indicates squareroot-transformed values of bacteria at genus level. The bacteric phylogenetic trees were indicated using neighbourjoining method and the relation among different samples was shown by Bray distance.

At the genus level, hierarchically clustered heatmap analysis (according to the profiles) indicated that the composition of digesta microflora of FM and RM groups shared similarity, while T0 and T1 were different (Figure 7).

### Relationship Between Microbial Community Structure and Dietary Characteristics

**Figure 8:**
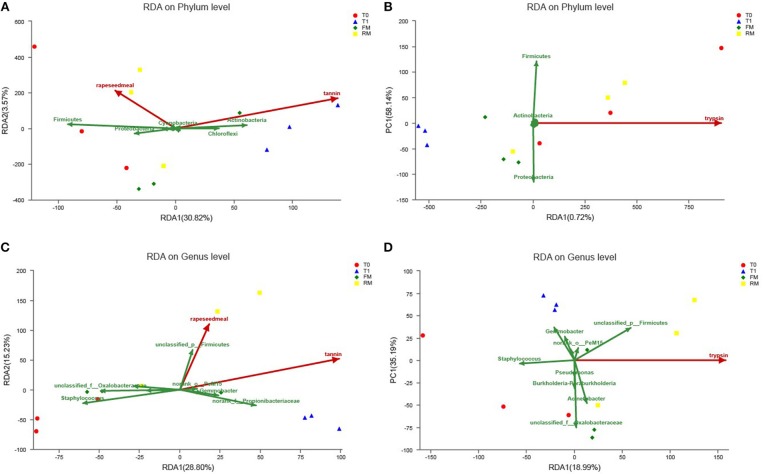
Redundancy analysis (RDA) of MiSeq data (symbols) and diet characteristics (arrows) (*n* = 3). Data of different groups were showed RDA of phylum **(A,B)** and genus **(C,D)**. **(A)** The RDA at phylum with rapeseed and tannin. **(B)** The RDA at phylum with trypsin. **(C)** The RDA at genus with rapeseed and tannin. **(D)** The RDA at genus with trypsin. The green arrows indicated intestinal microbiota, red arrows indicated diet characteristics and some biochemical indexes, the length of red arrows indicated the influence on intestine microbiota; the angle of red arrows and green arrows indicated correlation (acute angle, positive correlation; obtuse angle, negative correlation; right angle, no correlation); project from sample to red arrows, the distance indicated the influence of diet characteristic on the sample.

The potential impacts of different factors, including dietary tannin, rapeseed meal, and several biochemical factors on the phylogenetic composition of digestive microbiota were investigated. RDA indicated the potential impacts of different environmental factors on the phylogenetic composition of the intestine microbiota. After removal of redundant variables, two environmental characteristics were chosen for RDA. As shown in Figure 8, tannin content (*P* = 0.003) and rapeseed meal content (*P* = 0.014) significantly affected the microbial community structure, and were related to the intestinal trypsin activity (*P* = 0.015).

The present study indicated that at the phylum level, tannin correlated positively with the relative abundance of Actinobacteria and Chloroflexi, and correlated negatively with the relative abundance of Firmicutes and Proteobacteria (Figure 8A), similar relationship was not obtained in rapeseed meal (Figure 8B). Rapeseed meal correlated positively with the relative abundance of Firmicutes and Proteobacteria, and correlated negatively with the relative abundance of Actinobacteria and Chloroflexi. At the genus level, tannin and rapeseed meal both correlated positively with the relative abundance of *PeM15, Propionibacteriaceae*, and *Gemmobacter*, and correlated negatively with the relative abundance of *Acinetobacter, Oxalobacteraceae*, and *Staphylococcus* (Figure 8C). The intestinal trypsin activity correlated positively with Firmicutes, *Acinetobacter*, and *Caldilineaceae* and correlated negatively with *Gemmobacter, Propionibacteriaceae*, and *Staphylococcus* (Figure 8D).

## Discussion

### Effect of Tannin and Rapeseed Meal on Growth and Allochthonous Intestinal Microbiota of the Grass Carp

In previous studies, negative effects were observed when rapeseed meal was supplemented in excess to aquaculture diets. Webster et al. (19) reported that the growth of channel catfish decreased when their diet contained 48% rapeseed meal. A similar result was also reported for Ussuri catfish, which showed that when 20% dietary fishmeal was replaced with rapeseed meal, the specific growth rate decreased (20). In the present study, the weight gain rate decreased in the RM group, while no negative effect was observed on growth performance in the T1 group. This indicates that dietary tannins were not the immediate reason for the 50% dietary rapeseed meal decreasing growth performance of the grass carp.

Although the weight gain rate was not influenced in T1, the FC ratio and feeding rate increased. This is consistent with reports on the Indian major carp, which could tolerate 2% dietary tannin without obvious effects on growth performance (37). However, when 2.5% dietary hydrolysable tannin was added to the diet of the Nile tilapia, the weight gain rate decreased and the FC ratio increased (38). It is generally accepted that tannin inhibit absorption by binding with dietary nutrition and forming indigestible complexes. Fish may increase their feed intake to alleviate poor nutrition, and the high FC ratio in T1 also corroborated this (25, 45).

Proteobacteria and Firmicutes are the most ubiquitous and common phyla of the intestinal microbiota of the grass carp (46, 47). In the present study, Proteobacteria, Firmicutes and Actinobacteria were identified as the dominant allochthonous intestinal microbiota in grass carp. This is consistent with the results of feeding grass carps with ryegrass, which is a widely used feed supplement in grass carp culture (46). However, tannin decreased the relative abundance of Proteobacteria and Firmicutes while it increased the relative abundance of Actinobacteria. Rapeseed meal decreased the relative abundance of Proteobacteria while it increased the relative abundance of Firmicutes and Actinobacteria. This suggests a relationship between growth performance, nutrition deposition, and intestinal microbiota especially Firmicutes.

### The Effect of Tannin and Rapeseed Meal on the Protein Metabolism of the Grass Carp

It is widely accepted that growth is associated with the deposition of nutrients, especially protein in fish (48). Numerous studies showed that dietary rapeseed meal influences the metabolism of protein (20, 49). In a study of the cobia (*Rachycentron canadum*), the muscle protein content, apparent digestibility coefficient of crude protein, nitrogen retention and protein efficiency ratio decreased when 13% dietary rapeseed meal was supplemented (50). Similar results were also reported for the rainbow trout (*Oncorhynchus mykiss*) and hybrid tilapia (*Oreochromis niloticus* × *Oreochromis aureus*) when 50% and 28.5% rapeseed meal was included into their diet, respectively (51, 52). Information about the effect tannin on the protein metabolism is limited in aquatic animals. Buyukcapar et al. (38) showed that the muscle protein content decreased with increasing dietary tannin levels in the Nile tilapia. However, Omnes et al. (53) suggested that the apparent digestibility coefficient of protein significantly decreased with dietary tannin levels in European seabass. In the present study, the muscle protein contents in the FM and T0 groups were higher than in the other treatments, indicating that 50% dietary rapeseed meal decreased protein deposition, and tannin application maybe an important reason. However, to test this hypothesis, additional studies are required.

Tannins have been reported to inhibit amino acid absorption *in vitro* by binding with trypsin and dietary proteins (54). However, in the present study, the activity of trypsin was significantly higher in T1 and RM groups compared to the other treatments. Previous studies showed that tannins impair protein digestion by forming less digestible complexes with dietary proteins rather than inhibiting digestive enzymes (25). This is because before dietary tannins were exposed to digestive enzymes, they could complex with saliva-specialized tannin-binding proteins and dietary proteins (55). Consequently, to increase the protein digestion ability, the hepatopancreas secretes more trypsin. Despite this compensation, there is a possibility that the body is still short of amino acids to synthesize new proteins, resulting in lower protein deposition. The low activities of hepatic AST and ALT in T1 also corroborate this.

In the hepatopancreas, aminotransferases catabolize amino acids, which are then absorbed in the intestine and transfer amino groups to alpha-keto acids (17). If faced with deficiency of the available amino acids, keto-acids decrease, and lower activities of AST and ALT are required (50). In the present study, significantly reduced activities of hepatic AST and ALT were found in T1 and RM groups compared with the other treatments. This indicated that the biosynthesis of protein decreased, and the deficient amino acid as a result of tannins and the unbalanced amino acids of rapeseed meal may be important. A similar result was found in Japanese seabass (*Lateolabrax japonicus*) fed with a diet contained 10% canola meal. The activities of ALT and AST in liver were affected, and the apparent digestibility coefficient values of dry matter, protein, lipid, and phosphorus decreased (17). Except antinutritional function, tannin has been suggested toxic to some animals (56). Injecting tannins (10 mg/kg body) into the common carp (*Cyprinus carpio* L.), indicated toxicity to oxidative stress induction and antioxidant enzyme inhibition (57). In rats, a single subcutaneous injection of tannins at 700 mg/kg body weight caused a significant breakdown of polyribosomes in the liver and inhibited the incorporation of amino acids into hepatic proteins (25). In the present study, the content of urea nitrogen in serum was lower in T1 and RM groups compared with other treatments. The albumin level was lower in the RM group than in the FM group, while serum globulin and hepatic MDA were higher in T1 and RM groups. This indicated that tannins decreased the available amino acids absorbed from the intestine and potentially damaged hepatopancreas health (58).

In addition to tannins, other antinutritional factors in rapeseed meal also exert negative effect on the protein metabolism (59). Phytic acid and glucosinolate in rapeseed meal have been reported to adversely affect the digestibility of protein, the bioavailability of amino acids, and the protein quality of diets (60). Indigestible phytic acid-protein complexes and phytic acid-mineral complexes were reported to decrease growth and protein digestibility of the rainbow trout (61). Glucosinolate breakdown products impede thyroid hormone metabolism and depress nutrition metabolism (62). A deleterious effect of isolated isothiocyanates on the protein digestive utilization, combined with thyroid disturbances, has been reported for the common carp (63).

Furthermore, an unbalance of amino acids is likely a central reason for the poor growth and low muscle protein content of the RM group (64). Essential amino acids (EAA), especially lysine (Lys) and methionine (Met), are generally limited in rapeseed meal (65). EAA deficiency could affect the absorption, synthesis, and metabolism of nutrients (50, 66). Enami (67) reported that EAA deficiency negatively affects the growth of fish fed with high RM-inclusion diets.

In the present study, the relative abundance of Firmicutes and Bacteroidetes decreased, while those of Cyanobacteria, Actinobacteria, Saccharibacteria, and Chloroflexi increased in group T1 compared to group T0. The relative abundance of Proteobacteria decreased in RM compared to FM, while Firmicutes increased, indicating that supplementation with tannins and rapeseed meal modified the bacterial community. Intestinal microbiota are closely associated with the nutritional metabolism, and could provide nutrition such as amino acids and peptidases for the host (4, 68, 69). Metagenomic data indicated that microbially mediated mechanisms of protein breakdown occur in the fish intestine, and enzyme-producing bacteria commonly play a significant role in grass carp (70–72). The genera *Streptococcus, Vibrio, Cetobacterium, Clostridia, Shewanella*, and *Neisseria* are well known protease-producing bacteria commonly found in the fish intestine (73–76). In the present study, the relative abundances of *Cetobacterium, Streptococcus*, and *Clostridia* were higher in the RM group compared to the FM group, which may be a compensatory effect to the unbalanced amino acid profile of the RM diet. However, the relative abundance of *Cetobacterium, Shewanella, Streptococcus, Clostridia*, and *Neisseria* decreased in the T1 group compared to the T0 group, which partly explains the negative effect of tannins on protein digestion. In this context, the bactericidal efficacy of tannins may be an important reason (77). *Caldilineaceae* are widely used in domestic wastewater treatment, since they play an important role in nitrogen removal (78). In the present study, *Caldilineaceae* was positively correlated with trypsin activity, which partly explains the relationship among diets, digestive enzyme and intestinal microbiota. *Gemmobacter* are α-Proteobacteria, which are widely distributed in natural and artificial environments and are used as denitrifying bacteria. They have been reported to decrease the absorption of protein (79). The relative abundance of *Gemmobacter* was higher in the T1 group than in the T0 group, and was negatively correlated with trypsin activity. This also indicated the negative effect of tannins on protein absorption.

### The Effect of Tannins and Rapeseed Meal on the Carbohydrate Metabolism of the Grass Carp

Carbohydrates are relatively inexpensive and a readily available source of energy for aquatic organisms, especially for herbivorous fish with their higher amylase activity, and metabolic enzyme activity (80, 81). After carbohydrates are absorbed, glycogen is mainly deposited in muscle and liver tissues (82). In the present study, the activity of amylase and hepatic glycogen in T1 and RM groups were both significantly higher than in other treatments. These results may be because dietary tannin stimulates sympathetic pathways and adrenaline, and adrenaline promotes the expression of amylase (83). This has been suggested as a countermeasure against the adverse effects of tannins through the formation of tannin–amylase complexes (84, 85). The activity of amylase increased, and the metabolism of carbohydrate was also influenced. A previous study indicated that 0.05% dietary α-amylase improved the carbohydrate metabolism of the grass carp and decreased lipid deposition, because α-amylase promotes the decomposition of carbohydrate to glucose, and more carbohydrates are used as energy but do not convert to lipids, which agrees with the results of the present study (86).

Therefore, dietary tannins may play an important role in the promotion of carbohydrate digestion and absorption, and amylase-producing bacteria may commonly be associated with this (87). Chloroflexi is a common bacterial phylum in sponges and contaminated marine sediment, and is very popular in treating municipal wastewater due to its carbohydrate degrading ability (88). Several *Propionibacteriaceae* can produce vitamin B_12_ and amylase (89). *Cetobacterium* is reported to ferment peptides and carbohydrates and produce vitamin B_12_ (90). In the present study, the relative abundances of Chloroflexi and *Propionibacteriaceae* were significantly higher in the T1 group than in the T0 group, and the relative abundance of *Cetobacterium* increased in the RM group compare to the FM group.

### The Effect of Tannin and Rapeseed Meal on the Lipid Metabolism of the Grass Carp

Lipids are a major non-protein energy source in fish diets. However, as a typical herbivorous fish, lipids are less available for the grass carp, and extensive lipid accumulation in practical culture occur frequently (91, 92). In the present study, the muscle lipid deposition and lipase activity decreased in T1 and RM groups compared to the other treatments. In a study on rats, 2.5% dietary hydrolysable tannin decreased the activity of lipase, and the large binding strength between tannin and lipase was suggested as the central reason (93). Therefore, this indicates that dietary rapeseed meal may decrease lipid deposition by suppressing dietary lipid absorption, and tannins may be the central reason.

A study on rats reported lower proportion of Bacteroides/Firmicutes in the intestine of obese rats. The reason was the mutually beneficial symbiotic relationship between Firmicutes and Bacteroides in intestine, which promoted energy absorption and storage in the host (94). This relationship was partly observed in the present study, since the ratios of the Bacteroides/Firmicutes were 10.69 and 12.43% in the intestine of fish that received semi-purified-diets, while the corresponding muscle fat ratios were 10.07 and 7.81%, respectively.

### The Effect of Tannins and Rapeseed Meal on the Health of Grass Carp Health

In dietary fishmeal replacement studies, oxidative stress and immune reactions were frequently reported (95, 96). In the present study, higher hepatic MDA and serum globulin levels were obtained in RM and T1 groups, and the toxicity of tannins may be partly responsible for this observation (57).

Intestinal bacterial diversity is an important indicator for intestinal health (2). The effect of dietary soybean proteins on the diversity of intestinal bacteria have been investigated in numerous studies (6, 97–99). According to the intermediate-disturbance hypothesis proposed by Connell (100), diversity is maximized at intermediate disturbance levels; therefore, a lower species diversity reflects a more stable microbial community. In the present study, the diversity of the allochthonous intestinal bacteria community in T1 and RM groups were higher compared with other treatments. This indicates that the intestinal environment was partly disturbed and the bacterial community was modified. However, the high diversity is the early stage of disruption and organisms may adapt and recover gradually (100).

Proteobacteria is a common dominant bacterium of the intestine of aquatic animals. Reveco et al. (98) reported enteritis in Atlantic salmon when 30% dietary fishmeal were replaced by SBM. In response to this change, the relative abundance of Proteobacteria decreased in the distal intestine. A study in humans showed a decrease in the relative abundance of Bacteroidotes and Firmicutes in patients with inflammatory bowel disease (101). In the present study, when 1.25% dietary tannins were supplemented, the relative abundances of Bacteroidotes, Firmicutes, and Proteobacteria decreased, and when 50% rapeseed meal was included in the diet of the grass carp, the relative abundant of Bacteroidotes and Proteobacteria decreased. This indicates that inclusion of 50% rapeseed meal in the grass carp diet may injure the intestine, and tannins may be the cause.

*Lactobacillus, Lactococcus, Weissella, Pseudomonas* and many *Bacillus* spp. (e.g., *B*. *megaterium, B*. *polymyxa*, and *B*. *subtilis*) have been examined as probiotics for aquaculture (46, 102–104). Lactic acid bacteria increase non-specific defense mechanisms against antigens, and are able to modulate the innate immune response by interacting with the intestinal epithelial barrier and stimulate the intestinal inflammatory immune response (98, 104). In the present study, the relative abundances of *Lactococcus, Lactobacillales, Weissella, Pseudomonas*, and *Bacillus* decreased in the T1 group compared to the T0 group, while *Pseudomonas* and *Bacillus* decreased in the RM group compared to the FM group. The lower abundance of probiotics may imply a less healthy fish intestine in fish fed with the diets T1 and RM (105, 106). However, the relative abundances of *Lactococcus* and *Latobacillales* increased in RM. This may be because plant ingredients contain higher levels of carbohydrates, which are preferentially used by lactic acid bacteria as substrate for growth (107). Despite their improved relative abundance, both types of bacteria were not sufficient to maintain the health of the grass carp.

## Conclusion

This experimental study showed that 1.25% tannin addition was not the main reason for the poor growth performance of grass carp induced by a diet containing 50% rapeseed meal. However, tannins impaired the protein metabolism, decreased the digestion and accumulation of lipids, and promoted carbohydrate digestion. Furthermore, tannins and rapeseed meal disturb the intestinal environment and alter its bacterial composition.

In future studies, more biomarkers would be examined to explain the underlying mechanisms of tannin and rapeseed meal on growth and health of grass carp, and more advanced technology such as metagenomic functional profiles should be applied to elucidate the relationship among bacterial community, host metabolic, disease phenotypes and diets.

## Data Availability Statement

The datasets generated for this study can be found in NCBI BioProject accession number PRJNA542992.

## Ethics Statement

The animal study was reviewed and approved by Institutional Animal Care and Use Committee (IACUC), Shanghai Ocean University (SHOU).

## Additional Information

Sequence information: all sequence data have been deposited in the NCBI Sequence Read Archive under accession code PRJNA542992.

## Author Contributions

Six authors are justifiably credited with authorship. JY, XH, and PC conceived the study and designed the experiments. JY, GZ, and ZH contributed to performing the experiment. JY and PC did the data analysis and wrote the paper. XH and ER revised the manuscript.

### Conflict of Interest

The authors declare that the research was conducted in the absence of any commercial or financial relationships that could be construed as a potential conflict of interest.
