# Supplementary figures and images for: Effect of Diet Supplemented With Rapeseed Meal or Hydrolysable Tannins on the Growth, Nutrition, and Intestinal Microbiota in Grass Carp (Ctenopharyngodon idellus)
Source: Front Nutr. 2019 Sep 25;6:154. doi: 10.3389/fnut.2019.00154 (PMC6773801; doi:10.3389/fnut.2019.00154)

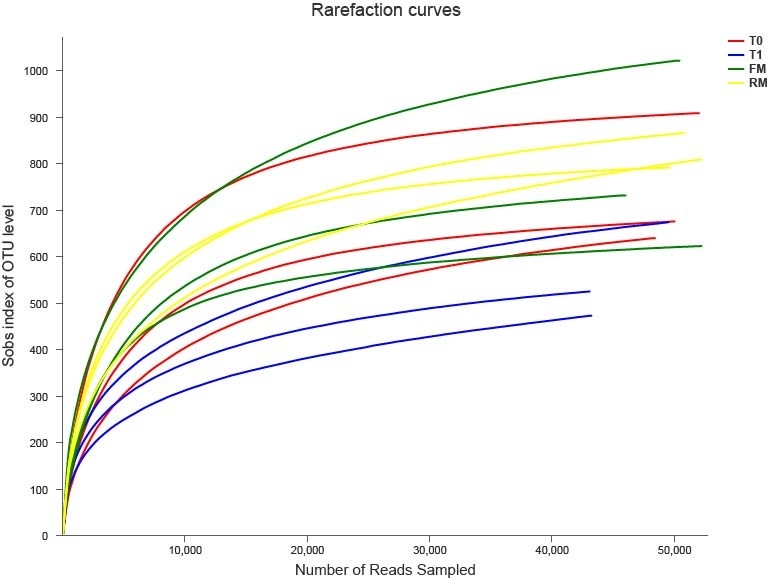

Supplement: Supplementary file 1 [file Image_1.JPEG]
